# Thermodynamic Stability of Fenclorim and Clopyralid

**DOI:** 10.3390/molecules27010039

**Published:** 2021-12-22

**Authors:** Ana R. R. P. Almeida, Bruno D. A. Pinheiro, Ana I. M. C. Lobo Ferreira, Manuel J. S. Monte

**Affiliations:** Centro de Investigação em Química (CIQUP), Department of Chemistry and Biochemistry, Faculty of Sciences, University of Porto, Rua do Campo Alegre, P-4169-007 Porto, Portugal; brunodapinheiro@gmail.com (B.D.A.P.); ana.ferreira@fc.up.pt (A.I.M.C.L.F.)

**Keywords:** fenclorim, clopyralid, vapor pressures, phase transitions, heat capacities, thermodynamic stability

## Abstract

The present work reports an experimental thermodynamic study of two nitrogen heterocyclic organic compounds, fenclorim and clopyralid, that have been used as herbicides. The sublimation vapor pressures of fenclorim (4,6-dichloro-2-phenylpyrimidine) and of clopyralid (3,6-dichloro-2-pyridinecarboxylic acid) were measured, at different temperatures, using a Knudsen mass-loss effusion technique. The vapor pressures of both crystalline and liquid (including supercooled liquid) phases of fenclorim were also determined using a static method based on capacitance diaphragm manometers. The experimental results enabled accurate determination of the standard molar enthalpies, entropies and Gibbs energies of sublimation for both compounds and of vaporization for fenclorim, allowing a phase diagram representation of the (*p*,*T*) results, in the neighborhood of the triple point of this compound. The temperatures and molar enthalpies of fusion of the two compounds studied were determined using differential scanning calorimetry. The standard isobaric molar heat capacities of the two crystalline compounds were determined at 298.15 K, using drop calorimetry. The gas phase thermodynamic properties of the two compounds were estimated through ab initio calculations, at the G3(MP2)//B3LYP level, and their thermodynamic stability was evaluated in the gaseous and crystalline phases, considering the calculated values of the standard Gibbs energies of formation, at 298.15 K. All these data, together with other physical and chemical properties, will be useful to predict the mobility and environmental distribution of these two compounds.

## 1. Introduction

**Figure 1:**
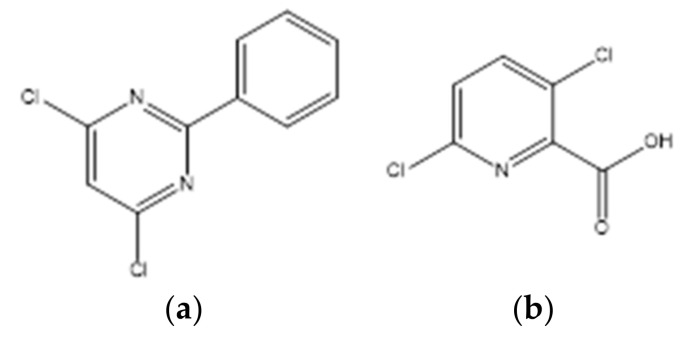
Structural formulas of the compounds studied in this work. (**a**) Fenclorim (4,6-dichloro-2-phenylpyrimidine); (**b**) Clopyralid (3,6-dichloro-2-pyridinecarboxylic acid).

Herbicides, also known as weed killers, are a broad class of pesticides that are used to control or manipulate undesirable vegetation, such as grasses and weeds, that may compromise the growth and profitability of crops [1]. They are efficient and cost-effective means of controlling nuisance plants when compared to hoeing, mowing, cultivation, or hand pulling and play a key role in farmland management and in reducing labor intensity [2,3,4]. While these chemicals help improve and rise crop yields, they also pose risks to the crops themselves which can be sensitive to these agents. To overcome this issue herbicide safeners have been developed to increase crop selectivity [5,6]. These are synthetic substances with the ability to protect grass crops from herbicide injury by a physiological or molecular mechanism, without reducing herbicidal activity on target weed species [3]. The safener fenclorim (Figure 1a) was designed to reduce the damage to rice (*Oryza sativa* L.) caused by herbicides belonging to the class of chloroacetanilides [7,8,9]. To ensure the safety of rice at an early stage, fenclorim is often formulated with pretilachlor [2-chloro-2′,6′-diethyl-*N*-(2-propoxyethyl)-acetanilide], which is one of the most widely used herbicides in rice-producing countries but poses a phytotoxicity risk for this cereal [7,9,10,11]. Fenclorim protects the rice from damage caused by pretilachlor mainly by speeding up the metabolism of this herbicide [7,9]. The pyridinecarboxylic acid herbicide clopyralid (Figure 1b) has been used effectively to control broadleaf weeds in pastures, turf, and in some agricultural crops such as barley, sugar beets, wheat, mint and oats [12]. It is a synthetic plant growth hormone that has some structural characteristics similar to those of natural hormones called auxins but is more persistent than these in plant tissue. This popular auxin-mimic type herbicide binds to molecules that are normally used as receptors for natural growth hormones, interfering with the normal growth of the plant and leading to its death in a short period of time [12,13,14]. Clopyralid is one of the systemic pesticides frequently found in drinking water [15]. Despite the harmful impact of these two compounds, some of their thermodynamic properties remain unknown, and therefore it was decided to perform a thermodynamic study of phase transition equilibria of fenclorim and clopyralid. The knowledge of properties related to the environmental distribution and mobility of this type of compounds as well as the evaluation of their thermodynamic stability, are essential information. Compound’s thermodynamic stability can be evaluated through the standard molar Gibbs energy of formation, $\Delta_{f}^{}G_{m}^{o}$. In addition to being an important property for calculating equilibrium constants of reactions, this function measures the thermodynamic tendency for a compound to decompose into its constituent elements, under standard state conditions. So, $\Delta_{f}^{}G_{m}^{o}$ of the crystalline and gaseous phases of fenclorim and clopyralid were determined in this work, at *T* = 298.15 K. As part of a broader study, this work aims to contribute to the knowledge of properties related to the transport, distribution, and environmental fate of compounds with harmful biological activity.

## *2.* Experimental Section

### 2.1. Materials

Table 1 reports detailed information on the purity and methods of purification and analysis of the two compounds studied. They were obtained commercially and further purified using sublimation under reduced pressure prior to the experimental determinations. The purity of the purchased compounds and of the purified samples were assessed by gas-liquid chromatography using an Agilent 4890D chromatograph equipped with a non-polar capillary HP-5 column, a flame ionization detector (FID) and using nitrogen as the carrier gas. The solvent used was dimethylformamide. The water content of the purified samples was determined using Karl Fischer coulometric titration. It was performed with a Methrom titration system, consisting of an 831 Coulometer (without diaphragm generator electrode and HYDRANAL™ as reagent). To determine the standard uncertainty of the measurements, four independent experiments were performed, and the error was assigned as the standard deviation. The specific densities of fenclorim and clopyralid were determined from the ratio mass/volume of three pellets of the compounds and are provided as Supplementary Materials, in Table S1. The relative atomic masses used in this work were those recommended by the IUPAC Commission in 2016 [16].

### 2.2. Thermal Analysis

The Hitachi-DSC7020 heat flow calorimeter was used to verify the absence of possible phase transitions in the crystalline phase of fenclorim and clopyralid and to determine their onset temperatures and enthalpies of fusion. Four independent runs were carried out using fresh samples (not melted before) sealed in airtight aluminum crucibles for each compound. The samples were scanned at 2.0 K^.^min^−1^ from *T* = 298.15 K to a temperature (20 to 25) K higher than their temperature of fusion under a controlled nitrogen flux that was used to avoid eventual contamination of the calorimeter. Calibration of the calorimeter was performed using the following high purity reference materials: benzoic acid (NIST SRM 39j), indium (Sigma-Aldrich, St. Louis, MO, USA, mass fraction >0.99999) and tin (Sigma-Aldrich, mass fraction >0.99999). The standard uncertainties of the calibration results are *u*(*T*/K) = 0.34 and *u*[$\Delta_{\mathrm{cr}}^{l}H_{m}^{o}(T_{\mathrm{fus}})$/kJ^.^mol^−1^] = 0.66. The onset temperatures of fusion, *T*_fus_, and molar enthalpies of fusion, $\Delta_{\mathrm{cr}}^{l}H_{m}^{o}(T_{\mathrm{fus}})$, determined in each run are reported in Table S2, together with the derived values of the molar entropies of fusion, $\Delta_{\mathrm{cr}}^{l}S_{m}^{o}(T_{\mathrm{fus}})$, and the available literature results.

### 2.3. Heat Capacity Drop Calorimetry

A high-precision heat capacity drop calorimeter [17,18,19,20] was used to measure the heat capacities of fenclorim and clopyralid, at *T* = 298.15 K. The calibration of the calorimeter was performed using sapphire (*α*-Al_2_O_3_ pellets, NIST-RM 720), $C_{p,m}^{o}$(*α*-Al_2_O_3_) = (79.03 ± 0.08) J·K ^−1^·mol^−1^ [21], based on a single drop temperature step (Δ*T* = 10.00 K) from *T*_i_ = 303.15 K to the final temperature *T*_f_ = 293.15 K. The accuracy of the calorimeter and methodology for the measurements of the heat capacities of crystalline and liquid compounds was evaluated based on the measurements of benzoic acid (Calorimetric Standard NIST 39j) and hexafluorobenzene as test substances [19,20,21,22,23], providing reliable high-quality heat capacity data for several compounds [24,25,26,27]. The standard isobaric molar heat capacity, $C_{p,m}^{o}$, at 298.15 K of each experiment for fenclorim and clopyralid, together with the mass of sample used in at least two independent series of drop experiments are presented in Table S3.

### 2.4. Vapor Pressure Measurements

#### 2.4.1. Knudsen Mass-Loss Effusion Method

The sublimation vapor pressures of fenclorim and clopyralid were measured at different temperatures using the Knudsen mass-loss effusion method. The apparatus used in this work allows the simultaneous operation of nine effusion cells contained in cylindrical holes inside three aluminum blocks, controlled at different temperatures. Three cells with different effusion orifice sizes are inserted in the holes of each block. The effusion orifices made by Goodfellow™ on platinum foil of (0.0125 ± 0.001) mm thickness, have the following areas: *A*_o_(A1) = *A*_o_(A2) = *A*_o_(A3) = (0.636 ± 0.004) mm^2^, *A*_o_(B1) = *A*_o_(B2) = *A*_o_(B3) = (0.785 ± 0.004) mm^2^, *A*_o_(C1) = *A*_o_(C2) = *A*_o_(C3) = (0.985 ± 0.004) mm^2^, where the uncertainties were calculated by the root sum square (RSS) method. The Clausing factors of the effusion orifices were calculated as *w*_o_ = 1/{1 + (*l*/2*r*)}, where *l* is the thickness of the platinum foil and *r* is the radius of the orifices, yielding the results 0.986, 0.988 and 0.989 for *w*_o_ of the orifices of the series A, B and C, respectively. Further details of this set-up, procedure and testing have been described before in detail [28].

In each effusion experiment, the mass of the sublimed sample, Δ*m*, was determined by weighing each effusion cell (±0.01 mg), before and after a convenient effusion time, *t*, in a system evacuated to a pressure near 1 × 10^−4^ Pa. At each temperature *T*, the vapor pressure *p* of the crystalline sample is calculated using Equation (1),

(1) $$p=\;\frac{\Delta m}{A_{o}w_{o}t}{\left(\frac{2\pi RT}{M}\right)}^{0.5}$$

where *M* is the molar mass of the effusing vapor and *R* is the molar gas constant (8.314462618 J^.^K^−1.^mol^−1^ [29]). The standard uncertainties of the temperatures and vapor pressure measurements were estimated as *u*(*T*/K) = 0.01 and *u*(*p*/Pa) = 0.02.

#### 2.4.2. Static Method Based on Capacitance Diaphragm Manometers

A static method was used to determine the vapor pressures of both crystalline and liquid phases (including supercooled liquid) of fenclorim (the limited amount of purified sample of clopyralid did not allow measurements of vapor pressure using this technique). This static apparatus is based on capacitance diaphragm gauges, previously tested and depicted in detail [30,31,32]. The capacitance diaphragm absolute gauge used in this work was obtained from MKS Instruments, Inc. and operate at self-controlled constant temperature: Baratron 631A11TBFP (*T*_gauge_ = 473 K) capable of measuring pressures over the range (0.5 to 2.6 × 10^2^) Pa and temperature of the condensed sample from (253 to 463) K. The uncertainty of the temperature measurements is estimated to be *u*(*T*/K) = ±0.01 and the expanded uncertainties (0.95 confidence level, *k* = 2) of the pressure measurements are adequately described by the expression *U*(*p*/Pa) = 0.01 + 0.0050 (*p*/Pa). Before starting the vapor pressure measurements, the samples are conveniently outgassed until repeated measurements at a selected temperature deliver consistent pressure results after escaping eventual traces of volatile impurities, including water.

### 2.5. Computational Chemistry Calculations

Standard ab initio molecular orbital calculations of fenclorim, clopyralid and all the auxiliary molecules considered in this work were performed with Gaussian 09 software package [33], using the G3(MP2)//B3LYP composite method [34]. This methodology is a variation of the Gaussian-3 (G3) theory [35] that uses the B3LYP density functional method for geometry optimization and zero-point energies. Information about that method is detailed described in the literature [34].

## 3. Results and Discussion

### 3.1. Thermodynamic Properties of Phase Transitions

The vapor pressures of the two compounds studied, measured using the effusion or the static methods, are listed in Table 2, where the effusion pressures are the mean of the results determined through the different effusion orifices at each temperature, presented in detail in Tables S4 and S5 (Supplementary Materials). The vapor pressures of the crystalline phase of fenclorim and clopyralid were measured, respectively, in the temperature ranges *T* = (311.1 to 333.2) K and *T* = (334.1 to 356.4) K, using the Knudsen mass-loss effusion technique. Moreover, the sublimation vapor pressures of fenclorim were measured in the temperature interval (326.0 to 365.5) K using a static method based on capacitance diaphragm manometers. This technique was also used to determine the vaporization vapor pressures of this compound in the temperature range *T* = (338.8 to 399.9) K. The truncated form of Clarke and Glew equation [36], Equation (2), was used to fit the experimental (*p*,*T*) data.

(2) $$R\ln\left(\frac{p}{p^{o}}\right)=-\frac{\Delta_{\mathrm{cd}}^{g}G_{m}^{o}\left(\theta \right)}{\theta }+\Delta_{\mathrm{cd}}^{g}H_{m}^{o}\left(\theta \right)\left(\frac{1}{\theta }-\frac{1}{T}\right)+\Delta_{\mathrm{cd}}^{g}C_{p,m}^{o}\left(\theta \right)\left[\left(\frac{\theta }{T}\right)-1+\ln\left(\frac{T}{\theta }\right)\right]$$

In this equation, *p*^o^ is a selected reference pressure (*p*^o^ = 10^5^ Pa in this work), *p* is the vapor pressure at the temperature *T*, *θ* is a reference temperature (in this work, unless stated otherwise, *θ* = 298.15 K), *R* is the molar gas constant and $\Delta_{\mathrm{cd}}^{g}G_{m}^{o}$, $\Delta_{\mathrm{cd}}^{g}H_{m}^{o}$ and $\Delta_{\mathrm{cd}}^{g}C_{p,m}^{o}$ are thermodynamic properties of sublimation or vaporization (respectively, the standard Gibbs energy, the enthalpy and the isobaric heat capacity).

Table 3 reports, for each compound, results of those properties and of $\Delta_{\mathrm{cd}}^{g}S_{m}^{o}$ (calculated using Equation (3)) and their related uncertainties for three different temperatures (*θ* = 298.15 K, *θ* = mean temperature of the experiments and *θ* = temperature of the triple point, for fenclorim). The vapor pressures calculated from Equation (2) for the three different temperatures are also reported in this table.

(3) $$\Delta_{cd}^{g}S_{m}^{o}(\theta )=\;\frac{\Delta_{cd}^{g}H_{m}^{o}(\theta )-\Delta_{cd}^{g}G_{m}^{o}(\theta )}{\theta }$$

If experimental sublimation or vaporization vapor pressures are determined over a wide temperature range (~50 K), the fit of Equation (2) to the experimental (*p*,*T*) data frequently yields accurate values of $\Delta_{cd}^{g}C_{p,m}^{o}(\theta )$. In this work, the values of $\Delta_{cr}^{g}C_{p,m}^{o}(\theta )$ and $\Delta_{l}^{g}C_{p,m}^{o}(\theta )$ were derived directly from the linear regression of Equation (2) to the crystalline and liquid experimental results of fenclorim, respectively. The sublimation results, determined through Knudsen effusion and static methods, were fit together using Equation (2), enabling to derive a reliable value of $\Delta_{cr}^{g}C_{p,m}^{o}(\theta )$. Since the sublimation temperature range considered in this work for clopyralid (using Knudsen effusion method) was not large enough, the value of $\Delta_{cr}^{g}C_{p,m}^{o}(\theta )$ could not be derived for this compound by such procedure. As an alternative, it was calculated as $\Delta_{cr}^{g}C_{p,m}^{o}(\theta )=C_{p,m}^{o}(g)-C_{p,m}^{o}(cr)$, where $C_{p,m}^{o}(g)$ and $C_{p,m}^{o}(cr)$ are, respectively, the gas and crystalline isobaric molar heat capacities. The values of $C_{p,m}^{o}(cr)$, determined through heat capacity drop calorimetry, and the derived standard isobaric specific heat capacities, $c_{p}^{o}$, are reported in Table 4, together with the specific densities and the volumetric heat capacities, $C_{p,m}^{o}$/V_m_, determined at *T* = 298.15 K for each compound. The experimental results of $C_{p,m}^{o}\left(\mathrm{cr}\right)$ are in agreement with the ones estimated using the group contribution values proposed by Acree Jr. and Chickos [37], also listed in this table.

The values of $C_{p,m}^{o}(g)$, determined at the temperature 298.15 K for the two compounds, were derived from statistical thermodynamics, calculated by means of the Gaussian 09 software package [33], using the vibrational frequencies from G3(MP2)//B3LYP calculations (scaled by a factor of (0.960 ± 0.022) [39]). The results of $C_{p,m}^{o}$(g, 298.15 K)/J·K^−1^·mol^−1^ for fenclorim and clopyralid were, respectively, (190.8 ± 5.7) and (153.8 ± 4.6). The standard uncertainties in $C_{p,m}^{o}(g)$ were estimated as *u*[$C_{p,m}^{o}$(g)] = 0.03·$C_{p,m}^{o}$(g) [40]. As the values of $C_{p,m}^{o}(cr)$ were determined at 298.15 K it was assumed that $\Delta_{cr}^{g}C_{p,m}^{o}(\theta )$ is approximately constant inside the assigned uncertainties. For fenclorim, the value $\Delta_{cr}^{g}C_{p,m}^{o}(298.15\;K)$ = −26.4 ± 13.3) J^.^K^−1^^.^mol^−1^, derived from the fitting of Equation (2) to the sublimation (*p*,*T*) data, is in agreement with the one calculated through the theoretical value of $C_{p,m}^{o}(g)$ and the experimental result of $C_{p,m}^{o}(cr)$, $\Delta_{cr}^{g}C_{p,m}^{o}(298.15\;K)$ = −23.5 ± 5.8) J^.^K^−1^^.^mol^−1^.

The average of the onset of fusion temperatures, *T*_fus_, molar enthalpies, $\Delta_{\mathrm{cr}}^{l}H_{m}^{o}(T_{\mathrm{fus}})$, and entropies, $\Delta_{\mathrm{cr}}^{l}S_{m}^{o}(T_{\mathrm{fus}})$, of the two compounds studied are reported in Table 5, together with the fusion properties of fenclorim derived indirectly through static vapor pressure measurements.

**Figure 2:**
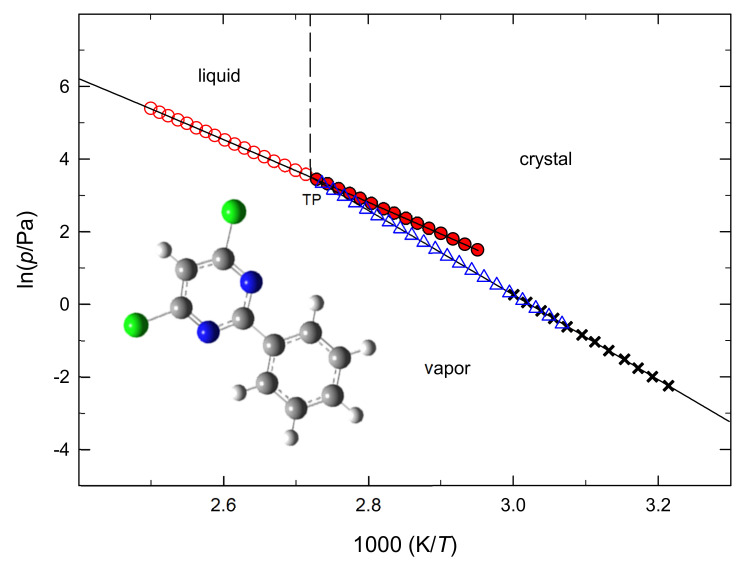
Phase diagram of fenclorim. ○, vaporization; ●, vaporization (supercooled liquid); Δ, sublimation (static method); **x**, sublimation (mean values of Knudsen effusion vapor pressures). Triple point data determined in this work: *T* = 367.4 K; *p* = 32.9 Pa.

**Figure 3:**
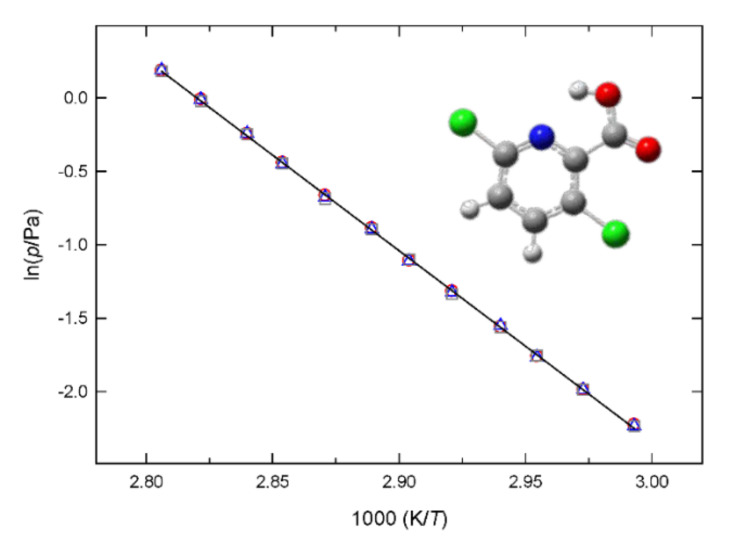
Plot of ln*p* against 1/*T* for clopyralid. ○, small effusion orifices; Δ, medium effusion orifices and □, large effusion orifices.

Figure 2 presents the phase diagram of fenclorim in the neighborhood of the triple point and Figure 3 shows the plot of vapor pressures against the reciprocal temperatures of clopyralid. To the best of our knowledge no (*p*,*T*) study was reported before for the two compounds studied in this work.

### 3.2. Thermodynamic Stability of Fenclorim and Clopyralid

The standard molar Gibbs energy of formation, $\Delta_{f}^{}G_{m}^{o}$, may be used to evaluate the thermodynamic stability of a compound at standard conditions. The values of this property are quite important to obtain equilibrium constants of reactions where they act as reagents or products. Therefore, the values of $\Delta_{f}^{}G_{m}^{o}$ of fenclorim and clopyralid were calculated in this work, at *T* = 298.15 K, in crystalline and gaseous phases. The results of the gas phase were calculated using Equation (4) and those of the crystalline phase were obtained by subtracting from $\Delta_{f}^{}G_{m}^{o}(g,298.15\;K)$ the results of $\Delta_{\mathrm{cr}}^{g}G_{m}^{o}$(298.15 K), derived through static and/or Knudsen effusion measurements.

(4) $$\Delta_{f}^{}G_{m}^{o}(298.15\;K,\;g)=\Delta_{f}^{}H_{m}^{o}(298.15\;K,\;g)-298.15\cdot \Delta_{f}^{}S_{m}^{o}(298.15\;K,\;g)$$

#### 3.2.1. Thermodynamic Properties of Formation in Gaseous Phase

Theoretical gas phase standard molar enthalpies of formation of fenclorim and clopyralid, $\Delta_{f}^{}H_{m}^{o}(g)$, were calculated from a set of hypothetical working reactions that involve maximum bonding pattern similarity between the products and the reactants. They also satisfy the requirement that accurate experimental data for all the auxiliary molecules used are reported in the literature. These reactions consider the enthalpies of reaction, $\Delta_{r}^{}H(298.15\;K)$, obtained through computational absolute standard enthalpies, $H_{298.15\;K}^{o}$, and the experimental gas phase enthalpies of formation of all included molecules, available in the literature. The G3(MP2)//B3LYP absolute enthalpies and the gas phase enthalpies of formation, at *T* = 298.15 K, for all the molecules considered in this work are provided as Supplementary Materials, in Table S6. The gas phase hypothetical reactions, the calculated values for the enthalpy variations as well as the resulting estimates of the enthalpies of formation in the gas phase, at *T* = 298.15 K, are reported in the Table S7 and Table S8, for fenclorim and clopyralid, respectively.

The gas phase standard molar entropies of formation, $\Delta_{f}^{}S_{m}^{o}(g)$, were calculated from the gas phase standard absolute entropies, $S_{m}^{o}(g,\;298.15\;K)$, obtained by G3(MP2)//B3LYP composite method, and from the following reference standard entropy values [41]: $S_{m}^{o}$[C(graphite)] = 5.740 J^.^K^−1.^mol^−1^, $S_{m}^{o}$(H_2_,g) = 130.680 J^.^K^−1.^mol^−1^, $S_{m}^{o}$(Cl_2_,g) = 223.079 J^.^K^−1.^mol^−1^, $S_{m}^{o}$(N_2_,g) = 191.609 J^.^K^−1.^mol^−1^ and $S_{m}^{o}$(O_2_,g) = 205.147 J^.^K^−1.^mol^−1^ (for clopyralid).

Considering the estimated results of $\Delta_{f}^{}H_{m}^{o}(g,\;298.15\;K)$ and $\Delta_{f}^{}S_{m}^{o}(g,\;298.15\;K)$, the values of $\Delta_{f}^{}G_{m}^{o}(g,\;298.15\;K)$ of fenclorim and clopyralid reported in Table 6 were determined using Equation (4).

#### 3.2.2. Thermodynamic Properties of Formation in Crystalline Phase

Considering the values of $\Delta_{f}^{}G_{m}^{o}(\mathrm{cr},\;298.15\;K)$ and of $\Delta_{f}^{}S_{m}^{o}(\mathrm{cr},\;298.15\;K)$, the standard molar enthalpy of formation in crystalline phase, $\Delta_{f}^{}H_{m}^{o}(\mathrm{cr},\;298.15\;K)$, was also estimated for both compounds. The gas phase standard molar entropies of formation, $\Delta_{f}^{}S_{m}^{o}(\mathrm{cr})$ were determined from the values of $S_{m}^{o}(\mathrm{cr},\;298.15\;K)$—calculated considering the $\Delta_{\mathrm{cr}}^{g}S_{m}^{o}(298.15\;K)$ results—and from the reference standard entropy values presented above.

Table 6 reports the gas phase standard entropies of the two nitrogen heterocycles and the values of their standard molar enthalpies, entropies and Gibbs energies of formation, in crystalline and gaseous phases, as well as the results of the sublimation properties derived through the vapor pressure measurements.

**Figure 4:**
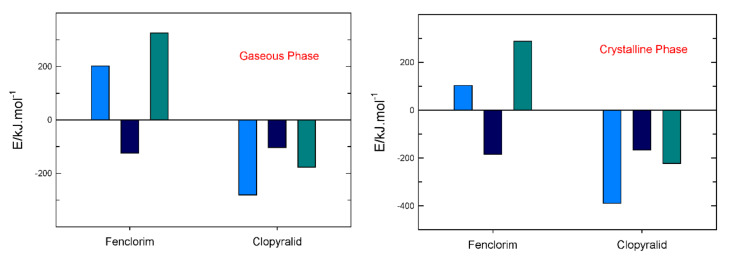
Relation between $\Delta_{f}^{}H_{m}^{o}$, $T\Delta_{f}^{}S_{m}^{o}$ and $\Delta_{f}^{}G_{m}^{o}$ for fenclorim and clopyralid in both gaseous and crystalline phases. **•** E **=**$\Delta_{f}^{}H_{m}^{o}$; **•** E **=**$T\Delta_{f}^{}S_{m}^{o}$; **•** E **=**$\Delta_{f}^{}G_{m}^{o}$.

The results reported in Table 6 (and shown in Figure 4) indicate that clopyralid is thermodynamically more stable―lower values of $\Delta_{f}^{}G_{m}^{o}$―than fenclorim in both crystalline and gaseous phases. It is easily noticeable that the enthalpic values of clopyralid have a major contribution to the smaller values of $\Delta_{f}^{}G_{m}^{o}$ of this compound in both phases.

**Figure 5:**
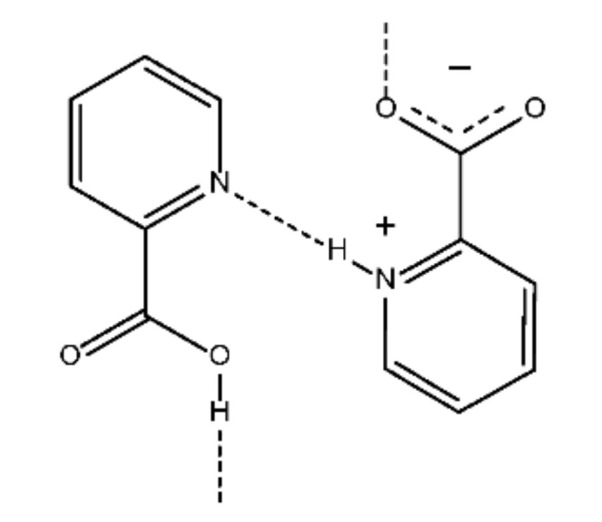
Schematic representation of the crystalline packing arrangement of 2-pyridinecarboxylic acid (adapted from ref. [42]).

The dominant enthalpic influence in the crystalline phase of clopyralid seems to be a consequence of the eventual O-H^⋯^O and N-H^⋯^N hydrogen bonds that are probably formed between clopyralid molecules. Although the crystal structure of this compound was not reported in the literature, we guess, as a credible hypothesis, that those intermolecular hydrogen bonds are formed in the crystalline phase of clopyralid similarly to the intermolecular bonds noticeable in the crystalline pattern of 2-pyridinecarboxylic acid [42], schematically represented in Figure 5. In the crystal structure of this compound, a zigzag chain is formed by the hydrogen bonds, N—H^⋯^N and O—H^⋯^O, where the protons are displaced around the twofold axis, or the center of symmetry and their site occupation factors were assumed to be 50% [42]. The authors of the article [42] concluded that the intermolecular N—H^⋯^N hydrogen bond is asymmetric and hydrogen atom is trapped at one of the two nitrogen atoms. Those bonds are much stronger than the CH^…^N hydrogen bonds and the C-Cl^⋯^π and π-π interactions present in the crystalline structure of fenclorim reported in the literature [38].

The stability of clopyralid in the gaseous phase hints the possible occurrence of an intramolecular hydrogen bond O-H^...^N in the isolated molecules of this compound. This bond should occur due to the proximity and orientation of the hydrogen of the hydroxyl group toward the nitrogen of the pyridine ring, with a distance of 1.95 × 10^−10^ m. This kind of hydrogen bond can be estimated as the difference in energy between the most stable conformation and the corresponding conformation optimized in a trans orientation relative to the proton acceptor fragment [43,44]. In this way, we estimated the value 11.5 kJ^.^ mol^−1^ for the O-H^…^N bond strength in this compound. This result is in close agreement with the ones reported in the literature for other substituted pyridine carboxylic acids: 14.0 kJ^.^mol^−1^, 11.8 kJ^.^mol^−1^, 12.4 kJ^.^mol^−1^, and 13.9 kJ^.^mol^−1^, respectively, for 2-pyridinecarboxylic acid, 2,4-pyridinedicarboxylic acid, 2,5-pyridinedicarboxylic acid, and 2,6-pyridinedicarboxylic acid [43].

## 4. Conclusions

The relevant conclusions of the present work are the following:

- The thermodynamic properties of fenclorim and clopyralid determined in this study contribute to the environmental impact assessment of these two compounds that have been used as pesticides.
- The temperatures and molar enthalpies of fusion of the two nitrogen heterocyclic compounds studied were determined using DSC and their crystalline isobaric molar heat capacities were measured, at 298.15 K, using heat capacity drop calorimetry. From these results the standard isobaric specific heat capacities and the volumetric heat capacities were obtained.
- The enthalpies, entropies and Gibbs energies of sublimation of both compounds and of vaporization of fenclorim were derived through vapor pressure measurements and the phase diagram representation of the (*p*,*T*) results, including triple point coordinates of this compound, is reported.
- Computational calculations at the G3(MP2)//B3LYP level were carried out, and the estimated gas-phase enthalpies of formation and absolute entropies of the two compounds were used to determine their Gibbs energies of formation in this phase. These results together with the sublimation ones enabled the calculation of the Gibbs energies of formation in crystalline phase.
- Considering the standard Gibbs energies of formation, it was concluded that clopyralid is thermodynamically more stable than fenclorim, in the crystalline and gaseous phases, mainly due to the enthalpic contribution.
- The standard molar enthalpy of formation in crystalline phase, $\Delta_{f}^{}H_{m}^{o}(\mathrm{cr},\;298.15\;K)$ was estimated for both compounds.
- It is probable that intramolecular OH^…^N hydrogen bonds occur in the gaseous phase of clopyralid and that intermolecular O-H^...^O and N-H^...^N hydrogen bonds exist between molecules of this crystalline compound, just like in the crystal structure of 2-pyridinecarboxylic acid.

## Figures and Tables

**Table 1:** Source, purity and methods of purification and analysis of the two compounds studied.

Compound	CASNR	Source	Minimum Initial Purity	Purification Method	Final Mass Fraction Purity	Analysis Method ^a^	Water Content ^b^ (%)
Fenclorim	3740-92-9	TCI	0.999 ^c^	Sublimation ^d^	0.9993	GC	0.02 ± 0.01
Clopyralid	1702-17-6	TCI	>0.98 ^e^		0.9989		0.04 ± 0.01

^a^ Gas-liquid chromatography with flame ionization detector (FID). ^b^ Determined using Karl Fisher coulometric titration. ^c^ Analysis certified by the manufacturer. ^d^ Under reduced pressure. ^e^ Minimum purity degree announced by the supplier.

**Table 2:** Vapor pressure results ^a^.

*T*/K	*p*/Pa	100Δ*p*/*p* ^b^	*T*/K	*p*/Pa	100Δ*p*/*p* ^b^	*T*/K	*p*/Pa	100Δ*p*/*p* ^b^
Fenclorim								
Crystalline phase (Knudsen effusion method) ^c^								
311.11	0.106	0.2	319.31	0.279	0.0	327.25	0.677	−0.5
313.29	0.136	−1.2	321.27	0.355	1.6	329.11	0.833	0.0
315.23	0.172	−0.8	323.10	0.430	0.1	331.27	1.051	0.0
317.10	0.220	1.8	325.28	0.538	−1.8	333.23	1.301	0.6
Crystalline phase (Static method)								
326.01	0.58	−1.1	339.81	2.54	0.8	353.58	9.67	0.8
327.95	0.72	−0.7	341.81	3.09	0.3	355.57	11.51	−0.2
329.90	0.89	−0.7	343.77	3.75	0.2	357.55	13.79	−0.3
331.91	1.11	−0.1	345.72	4.57	0.8	359.49	16.35	−0.8
333.91	1.37	−0.1	347.70	5.52	0.5	361.50	19.64	−0.4
335.88	1.69	0.3	349.70	6.69	0.5	363.49	23.32	−0.9
337.86	2.09	1.1	351.65	8.02	0.2	365.53	28.05	0.2
Liquid phase (Static method)								
338.80	4.40 ^d^	−0.1	360.41	20.97 ^d^	0.8	382.19	80.91	−0.4
340.84	5.13 ^d^	−0.3	362.38	23.91 ^d^	0.8	384.18	90.85	−0.4
342.76	5.95 ^d^	0.0	364.43	27.33 ^d^	0.7	386.23	102.8	0.2
344.71	6.92 ^d^	0.5	366.43	30.85 ^d^	−0.1	388.06	114.7	0.8
346.72	8.00 ^d^	0.2	368.43	35.37	0.7	390.07	126.6	−0.6
348.67	9.21 ^d^	0.1	370.36	39.54	−0.3	392.06	143.2	0.7
350.60	10.55 ^d^	−0.1	372.36	45.07	0.3	393.98	157.7	−0.1
352.60	12.18 ^d^	0.1	374.42	50.47	−1.1	396.07	177.0	0.1
354.43	13.72 ^d^	−0.8	376.30	57.21	0.0	397.94	194.6	−0.4
356.55	15.91 ^d^	−0.7	378.37	64.37	−0.7	399.88	217.0	0.2
358.53	18.29 ^d^	−0.2	380.22	72.49	0.2			
Clopyralid								
Crystalline phase (Knudsen effusion method) ^c^								
334.11	0.107	1.9	342.36	0.266	−1.3	350.40	0.640	−0.4
336.38	0.137	0.1	344.36	0.330	−1.6	352.13	0.783	1.4
338.46	0.172	−1.0	346.12	0.410	0.8	354.41	0.985	0.7
340.12	0.211	0.5	348.34	0.508	−1.6	356.38	1.203	0.4

^a^ The standard uncertainty of the temperature is *u*(*T/*K) = 0.01 and the expanded uncertainties (0.95 confidence level, *k* = 2) of the vapor pressures are *U*(*p*/Pa) = 0.01 + 0.0050 (*p*/Pa) for static pressures; *u*(*p*/Pa) = 0.02 for the effusion pressures. ^b^ Δ*p = p − p*_calc,_ where *p*_calc_ is calculated from the Clarke and Glew, Equation (2), with parameters given in Table 3. ^c^ The reported effusion pressures are the mean of the values obtained using the small, medium, and large effusion orifices. ^d^ Vapor pressures of the supercooled liquid.

**Table 3:** Standard (*p*^o^ = 10^5^ Pa) thermodynamic properties of sublimation and of vaporization of the compounds studied.

Δ*T*	*θ*	$\Delta_{\mathrm{cd}}^{g}G_{m}^{o}\left(\theta \right){\;}^{a}$	*p* ^b^	$\Delta_{\mathrm{cd}}^{g}H_{m}^{o}\left(\theta \right){\;}^{a}$	$\Delta_{\mathrm{cd}}^{g}S_{m}^{o}\left(\theta \right){\;}^{c}$	*R* ^2^	$-\Delta_{\mathrm{cd}}^{g}C_{p,m}^{o}\left(\theta \right){\;}^{a}$	*σ*_r_^d^
K	K	kJ^.^mol^−1^	Pa	kJ^.^mol^−1^	J^.^K^−1.^mol^−1^		J^.^K^−1.^mol^−1^	
Fenclorim								
Crystalline phase (Knudsen effusion method)								
311.1 to 333.2	298.15	38.19 ± 0.06	2.04 × 10^−2^	98.1 ± 0.8	200.9 ± 2.7	0.9998	23.5 ± 5.8 ^e^	0.0110
	322.17 ^f^	33.38 ± 0.02	3.87 × 10^−1^	97.6 ± 0.8				
Crystalline phase (static method)								
326.0 to 365.5	298.15	38.22 ± 0.03	2.01 × 10^−2^	98.1 ± 0.2	200.8 ± 0.7	1.0000	23.5 ± 5.8 ^e^	0.0064
	345.77 ^f^	28.74 ± 0.02	4.55	97.0 ± 0.2				
Crystalline phase (Knudsen effusion + static methods)								
311.1 to 365.5	298.15	38.18 ± 0.06	2.05 × 10^−2^	98.0 ± 1.1	200.6 ± 3.7	1.0000	26.4 ± 13.3 ^g^	0.0092
	338.32 ^f^	30.20 ± 0.01	2.17	96.9 ± 0.2				
	367.39 ^h^	24.50 ± 0.03	32.9	96.1 ± 0.2				
Liquid phase (static method) ^i^								
338.8 to 399.9	298.15	33.85 ± 0.10	1.17 × 10^−1^	76.1 ± 1.0	141.7 ± 3.4	1.0000	61.5 ± 7.1 ^g^	0.0053
	369.34 ^f^	24.25 ± 0.01	37.2	71.7 ± 0.1				
	367.39 ^h^	24.50 ± 0.01	32.9	71.9 ± 0.1				
Clopyralid								
Crystalline phase (Knudsen effusion method)								
334.1 to 356.4	298.15	45.82 ± 0.14	9.39 × 10^−4^	109.1 ± 1.0	212.2 ± 3.4	0.9998	21.5 ± 4.8 ^e^	0.0124
	345.24 ^f^	35.91 ± 0.02	3.69 × 10^−1^	108.1 ± 1.0				

^a^ Uncertainties are expressed as the expanded uncertainty (0.95 level of confidence, *k* = 2). ^b^ Calculated from Equation (2) for three different temperatures (*θ* = 298.15 K, *θ* = mean temperature of the experiments and *θ* = temperature of the triple point). ^c^ Calculated using Equation (3); uncertainties calculated through the RSS method. ^d^ *σ*_r_ is the relative standard deviation of the fit, defined as $\sigma_{r}={\left[\sum_{i=1}^{n}(ln\;p\;-\;ln\;p{\;}_{calc}){\;}_{i}^{2}/\;(n\;-m)\right]}^{1/2}$. ^e^ Calculated as $\Delta_{cr}^{g}C_{p,m}^{o}(\theta )=C_{p,m}^{o}(g)-C_{p,m}^{o}{\left(cr\right)}_{\exp}$. ^f^ Mean temperature. ^g^ Adjustable parameter derived from the fittings of Equation (2) to the (*p*,*T*) data. Uncertainties are standard deviations of the least-squares regressions. ^h^ Temperature of the triple point. ^i^ Including supercooled liquid.

**Table 4:** Experimental and estimated crystalline standard molar heat capacities $C_{p,m}^{o}$, specific heat capacities, $c_{p}^{o}$, densities and volumetric heat capacities, $C_{p,m}^{o}$ /*V*_m_, at *T* = 298.15 K of fenclorim and clopyralid.

Compound	$C_{p,m}^{o}{\;}^{a}$	Molar Mass	$c_{p}^{o}$	Density	$C_{p,m}^{o}/{V_{m\;}}^{b}$	$C_{p,m}^{o}(\mathrm{Estimated}){\;}^{c}$
	J^.^K^−1.^mol^−1^	g·mol^−1^	J·K^–1^·g^–1^	g^.^cm^−3^	J·K^–1^·cm^–3^	J·K^–1^·mol^–1^
Fenclorim	214.3 ± 1.2	225.074	0.952 ± 0.005	1.541 ^d^	1.467 ± 0.008	221.8 ± 17.0
Clopyralid	175.3 ± 1.2	191.999	0.913 ± 0.006	1.64 ± 0.05 ^e^	1.497 ± 0.050	181.4 ± 17.0

^a^ The reported experimental uncertainties are twice the standard deviation of the mean and includes the calibration uncertainty. ^b^ Calculated considering the specific heat capacities, $c_{p}^{o}$, and the experimental density values. ^c^ Estimated using the group contribution values proposed by Acree Jr. and Chickos [37]. ^d^ Ref. [38]. ^e^ Average of three measurements of the volume and mass of three pellets.

**Table 5:** Fusion properties: temperature, molar enthalpy and entropy of the compounds studied.

*T*_tp_/K	*T*_fus_/K ^a^	$\Delta_{cr}^{l}H_{m}^{o}{\left(T\right)}^{b}\;$/kj·mol^−1^	$\Delta_{cr}^{l}S_{m}^{o}(T{)}^{b,c}$/J·K^−1^·mol^−1^	Method/Ref.
Fenclorim				
	368.61 ± 0.35	23.08 ± 0.69 ^a^	62.6 ± 1.9	DSC/this work
367.39		24.2 ± 0.2		VP/this work
Clopyralid				
	422.63 ± 0.38	27.59 ± 0.67 ^a^	65.3 ± 1.6	DSC/this work

^a^ Standard uncertainty calculated through the RSS method combining the expanded uncertainties of the four experimental runs (0.95 level of confidence, *k* = 3.18) with the standard uncertainties of the DSC calibration. ^b^ *T* represents the temperature of fusion or the temperature of the triple point (*T*_tp_). ^c^ Uncertainties calculated through the RSS method.

**Table 6:** Standard (*p*^o^ = 10^5^ Pa) molar absolute entropies and standard molar enthalpies, entropies and Gibbs energies of formation and sublimation, at *T* = 298.15 K, of fenclorim and clopyralid.

		Fenclorim	Clopyralid
Gas phase	$\Delta_{f}^{}H_{m}^{o}{}^{a}\;$/kJ·mol^−1^	201.4 ± 2.9	−280.3 ± 1.7
	$S_{m}^{o}{}^{b}\;$/J·K^−1^·mol^−1^	447.6	407.7
	$\Delta_{f}^{}S_{m}^{o}\;$/J·K^−1^·mol^−1^	−416.5	−346.8
	$T\Delta_{f}^{}S_{m}^{o}\;$/kJ·mol^−1^	−124.2	−103.4
	$\Delta_{f}^{}G_{m}^{o}{}^{c}\;$/kJ·mol^−1^	325.6 ± 2.9	−176.9 ± 1.7
Thermodynamic Properties of Sublimation ^d^	$\Delta_{cr}^{g}H_{m}^{o}\;$/kJ·mol^−1^	98.0 ± 1.1	109.1 ± 1.0
	$\Delta_{cr}^{g}S_{m}^{o}$/J·K^−1^·mol^−1^	200.6 ± 3.7	212.2 ± 3.4
	$\Delta_{cr}^{g}G_{m}^{o}\;$/kJ·mol^−1^	38.2 ± 0.1	45.8 ± 0.1
Crystalline phase	$\Delta_{f}^{}H_{m}^{o}\;$/kJ·mol^−1^	103.4 ± 3.1	−389.4 ± 2.0
	$S_{m}^{o}{}^{e}\;$/J·K^−1^·mol^−1^	247.0 ± 3.7	195.5 ± 3.4
	$\Delta_{f}^{}S_{m}^{o}\;$/J·K^−1^·mol^−1^	−617.1 ± 3.7	−559.0 ± 3.4
	$T\Delta_{f}^{}S_{m}^{o}\;$/kJ·mol^−1^	−184.0 ± 1.1	−166.7 ± 1.0
	$\Delta_{f}^{}G_{m}^{o}{}^{f}\;$/kJ·mol^−1^	287.4 ± 2.9	−222.7 ± 1.7

^a^ The uncertainty assigned correspond to the expanded uncertainty determined from the estimated standard deviation of the mean (0.95 level of confidence) for the working reactions reported in SI (Tables S7 and S8), using k = 2.20 for fenclorim and k = 2.02 for clopyralid, respectively. ^b^ Obtained from G3(MP2)//B3LYP method for a frequency factor scale of 1.0029 [45]. ^c^ Calculated using Equation (4). ^d^ Derived through vapor pressure measurements. ^e^ Calculated from $S_{m}^{o}(298.15\;K,\;\mathrm{cr})=S_{m}^{o}(298.15\;K,\;g)-\Delta_{\mathrm{cr}}^{g}S_{m}^{o}(298.15\;K)$. ^f^ Calculated from $\Delta_{f}^{}G_{m}^{o}(298.15\;K,\;\mathrm{cr})=\Delta_{f}^{}G_{m}^{o}(298.15\;K,\;g)-\Delta_{\mathrm{cr}}^{g}G_{m}^{o}(298.15\;K)$.

## Data Availability

Not applicable.

## Supplementary Materials

The following supporting information can be downloaded, Table S1. Specific densities of fenclorim and clopyralid. Table S2. DSC results: temperatures, molar enthalpies and entropies of fusion of fenclorim and clopyralid. Table S3. Standard molar heat capacity results, at *T* = 298.15 K, for fenclorim and clopyralid. Table S4. Effusion vapor pressure results for crystalline fenclorim. Table S5. Effusion vapor pressure results for crystalline clopyralid. Table S6. Calculated absolute enthalpies, *H*, (in Hartree, Eh) of all considered molecules estimated using G3(MP2)//B3LYP. Literature values of $\Delta_{f}^{}H_{m}^{o}$(g). Table S7. Working reactions and computed enthalpies of reaction, $\Delta_{r}^{}H_{m}^{o}$, and formation, $\Delta_{f}^{}H_{m}^{o}$, of fenclorim in the gaseous phase, at *T* = 298.15 K. Table S8. Working reactions and computed enthalpies of reaction, $\Delta_{r}^{}H_{m}^{o}$, and formation, $\Delta_{f}^{}H_{m}^{o}$, of clopyralid in the gaseous phase, at *T* = 298.15 K.

## Sample Availability

Samples of the compounds are not available from the authors.
