# Supplementary material for: Thermodynamic Stability of Fenclorim and Clopyralid
Source: Molecules. 2021 Dec 22;27(1):39. doi: 10.3390/molecules27010039 (PMC8746602; doi:10.3390/molecules27010039)
Supplement: Supplementary file 1 [file molecules-27-00039-s001.zip › molecules-1494381-supplementary.pdf]

molecules

Supplementary Material

Thermodynamic Stability of Fenclorim and Clopyralid

Ana R. R. P. Almeida *, Bruno D. A. Pinheiro, Ana I.M.C. Lobo Ferreira and Manuel J. S. Monte *

Centro de Investigação em Química (CIQUP), Department of Chemistry and Biochemistry, Faculty of Sciences University of Porto, Rua do Campo Alegre, P-4169-007 Porto, Portugal; brunodapinheiro@gmail.com (B.D.A.P.); ana.ferreira@fc.up.pt (A.I.M.C.L.F.);
* Correspondence: ana.figueira@fc.up.pt (A.R.R.P.A.); mjmonte@fc.up.pt (M.J.S.M.)

This Supporting Material file contains the following contents:

- Specific densities of fenclorim and clopyralid
- DSC results: temperatures, molar enthalpies, and entropies of fusion of fenclorim and clopyralid
- Standard molar heat capacity results, $C^{o}_{p,\mathrm{m}}$, at T = 298.15 K for fenclorim and clopyralid
- Effusion vapor pressure results of fenclorim and clopyralid
- Computational study
- References

1. Specific Densities

Considering the agreement between the density value of fenclorim reported in the literature, 1.541 g.cm^{-3} [1], and the one determined experimentally in the present work for this compound, (1.43 ± 0.02) g.cm^{-3}, it was decided to perform the experimental determination of the density of clopyralid (3,6-dichloro-2-pyridinecarboxylic acid). The obtained result, (1.64 ± 0.03) g.cm^{-3}, was compared with the value published in the literature for the similar compound, 2-pyridinecarboxylic acid, 1.509 g.cm^{-3} [2].

Table S1. Specific densities of fenclorim and clopyralid .

Compound	Density / g.cm^{-3}
	1.44
**Fenclorim**	1.41
	1.44
Mean[a]	**1.43 ± 0.04**
Literature	1.541 [1]
	1.364 [3]
	1.4 ± 0.1 [4]
	1.5 (20 ºC) [5,6]
	1.62
**Clopyralid**	1.63
	1.67
Mean[a]	**1.64 ± 0.05**
Literature	1.5163 (60.17 ºC) [7]

1.6 ± 0.1 [4]

[a]Average of three measurements of the volume and mass of three pellets. The uncertainty assigned correspond to the expanded uncertainty determined from the standard deviation of the mean of the three measurements and the coverage factor $k = 4.30$ (0.95 level of confidence)

2. Differential Scanning Calorimetry

Table S2. DSC results: temperatures, molar enthalpies and entropies of fusion of fenclorim and clopyralid.

Exp.	T_{fus}(onset) / K	$\Delta_{cr}^{l}H_{m}^{o}(T_{fus})$ / kJ·mol⁻¹	$\Delta_{cr}^{l}S_{m}^{o}(T_{fus})$ / J·K⁻¹·mol⁻¹
		**Fenclorim**	
1	368.64	23.07	
2	368.67	23.08	
3	368.50	23.05	62.6 ± 1.9[b]
4	368.63	23.11	
Mean	368.61 ± 0.35[a]	23.08 ± 0.69[a]	
Lit.	365-366 [8,9]		
	370.1 [10-13]		
		**Clopyralid**	
1	422.65	27.46	
2	422.75	27.65	
3	422.68	27.58	65.3 ± 1.6[b]
4	422.45	27.69	
Mean	422.63 ± 0.38[a]	27.59 ± 0.67[a]	
Lit.	424-425 [14]		
	422.8 [15]		
	422-424 [16]		

[a]Standard uncertainty calculated through the RSS method combining the expanded uncertainties of the four experimental runs (0.95 level of confidence, $k = 3.18$) with the standard uncertainties of the DSC calibration. [b]Uncertainties calculated through the RSS method.

3. Heat Capacity Drop Calorimetry

Table S3. Standard molar heat capacity results, $C_{p,m}^{o}$, at $T = 298.15$ K for fenclorim and clopyralid. The calibration constant used to calculate the $C_{p,m}^{o}$ was derived from the sapphire [NBS, SRM 720, (α-Al_2O_3)] calibration, (6.6809 ± 0.0146 W·V⁻¹).

Exp	m_{sample}/g	N_{drop}	$\langle T_{furnace}\rangle$/K	$\langle T_{calorimeter}\rangle$/K	T/K	$C_{p,m}^{o}$/J·K⁻¹·mol⁻¹
			**Fenclorim**			
I	0.48467	11	303.10	293.17	298.14	214.6 ± 1.2
II	0.42311	36	303.08	293.18	298.13	214.1 ± 1.2
III	0.40059	10	303.11	293.17	298.14	214.6 ± 1.2
			**Clopyralid**			
I	0.39698	8	303.17	293.17	298.17	175.4 ± 1.4
III	0.39317	11	303.16	293.17	298.17	175.3 ± 1.0

N_{drop} = number of drop experiments; $T_{furnace}$ = average temperature of the furnace; $T_{calorimeter}$ = average temperature of the calorimeter; the uncertainty reported is twice the standard deviation of the mean and the calibration uncertainty is included.

4. Effusion Vapor Pressure Results

Table S4. Effusion vapor pressure results for crystalline fenclorim.[a.]

T/K [b]	*t*/s	Orifices	*m*/mg			*p*/Pa [b]				100Δ*p*/*p* [c]
			m_S	m_M	m_L	p_S	p_M	p_L	p_{mean}	
311.11	22000	A_1-B_4-C_7	5.48	6.71	8.35	0.107	0.106	0.105	0.106	0.2
313.29	22000	A_2-B_5-C_8	7.06	8.60	10.71	0.138	0.136	0.135	0.136	-1.2
315.23	22000	A_3-B_6-C_9	8.83	10.85	13.52	0.173	0.172	0.171	0.172	-0.8
317.10	15788	A_1-B_4-C_7	8.06	9.95	12.34	0.221	0.220	0.218	0.220	1.8
319.31	15788	A_2-B_5-C_8	10.27	12.19	16.00	0.282	0.271	0.283	0.279	0.0
321.27	15788	A_3-B_6-C_9	12.94	15.09	19.89	0.357	0.355	0.353	0.355	1.6
323.10	18303	A_1-B_4-C_7	18.30	22.27	27.65	0.436	0.430	0.425	0.430	0.1
325.28	18303	A_2-B_5-C_8	22.76	28.01	34.25	0.545	0.542	0.528	0.538	-1.8
327.25	18303	A_3-B_6-C_9	28.43	34.76	43.66	0.682	0.675	0.675	0.677	-0.5
329.11	10832	A_1-B_4-C_7	20.66	25.39	31.52	0.840	0.835	0.825	0.833	0.0
331.27	10832	A_2-B_5-C_8	25.88	31.54	40.15	1.056	1.041	1.055	1.051	0.0
333.23	10832	A_3-B_6-C_9	32.02	38.9	49.49	1.310	1.288	1.304	1.301	0.6

[a]Results related to the small (A_1, A_2, A_3), medium (B_4, B_5, B_6) and large (C_7, C_8, C_9) effusion orifices are denoted, respectively, by the subscripts *S*, *M* and *L*. [b]Estimated standard uncertainties: $u(T/\mathrm{K}) = 0.01$; $u(p/\mathrm{Pa}) = 0.02$. [c]$\Delta p = p - p_{calc.}$, where p_{calc} is calculated from the Clarke and Glew equation, Eq. (2).

Table S5. Effusion vapor pressure results for crystalline clopyralid.[a.]

***T*/K** [b]	***t*/s**	**Orifices**	***m*/mg**			***p*/Pa** [b]				**100Δ*p*/*p*** [c]
			m_S	m_M	m_L	p_S	p_M	p_L	p_{mean}	
334.11	23405	A_1-B_4-C_7	5.26	6.43	8.08	0.108	0.107	0.107	0.107	1.9
336.38	23405	A_2-B_5-C_8	6.66	8.24	10.29	0.137	0.137	0.137	0.137	0.1
338.46	23405	A_3-B_6-C_9	8.33	10.23	12.98	0.172	0.171	0.173	0.172	-1.0
340.12	21936	A_1-B_4-C_7	9.51	11.88	14.78	0.210	0.212	0.210	0.211	0.5
342.36	21936	A_2-B_5-C_8	12.09	14.81	18.45	0.268	0.266	0.263	0.266	-1.3
344.36	21936	A_3-B_6-C_9	14.84	18.27	23.17	0.330	0.329	0.332	0.330	-1.6
346.12	14792	A_1-B_4-C_7	12.46	15.28	19.23	0.412	0.409	0.409	0.410	0.8
348.34	14792	A_2-B_5-C_8	15.49	18.93	23.57	0.514	0.508	0.503	0.508	-1.6
350.40	14792	A_3-B_6-C_9	19.34	23.72	29.89	0.643	0.638	0.640	0.640	-0.4
352.13	14474	A_1-B_4-C_7	22.98	28.47	35.55	0.783	0.785	0.780	0.783	1.4
354.41	14474	A_2-B_5-C_8	28.96	35.72	44.35	0.990	0.988	0.977	0.985	0.7
356.38	14474	A_3-B_6-C_9	35.09	43.52	54.27	1.203	1.207	1.198	1.203	0.4

[a]Results related to the small (A_1, A_2, A_3), medium (B_4, B_5, B_6) and large (C_7, C_8, C_9) effusion orifices are denoted, respectively, by the subscripts *S*, *M* and *L*. [b]Estimated standard uncertainties: $u(T/\mathrm{K}) = 0.01$; $u(p/\mathrm{Pa}) = 0.02$. [c]$\Delta p = p - p_{calc.}$, where p_{calc} is calculated from the Clarke and Glew equation, Eq. (2).

5. Computational Study

Table S6. Calculated absolute standard enthalpies, $H^{\circ}_{298.15\mathrm{K}}$, (in Hartree, E_h)[a] of all considered molecules estimated using G3(MP2)/B3LYP. Literature values of $\Delta_f H^{o}_{m}$ (g). 1 a. u. (Hartree) corresponds to 2625.50 kJ.mol^{-1}.

Compound	Absolute Enthalpy / E_h	$\Delta_f H^o_m(g)$ / $kJ \cdot mol^{-1}$
Methane	-40.420547	-74.4 ± 0.4 [17]
Ethane	-79.651017	-83.8 ± 0.3 [17]
Benzene	-231.835164	82.9 ± 0.9 [18]
Chlorobenzene	-690.999276	52.0 ± 1.3 [17]
o-Dichlorobenzene	-1150.160000	30.2 ± 2.1 [17]
m-Dichlorobenzene	-1150.162189	25.7 ± 2.1 [17]
p-Dichlorobenzene	-1150.162055	22.5 ± 0.5 [17]
Biphenyl	-462.501235	180.3 ± 3.3 [18]
Pyridine	-247.873431	140.4 ± 0.7 [17]
2-Chloropyridine	-707.039096	104.5 ± 1.8 [19]
3-Chloropyridine	-707.036001	107.6 ± 1.7 [19]
2,3-Dichloropyridine	-1166.198732	89.5 ± 3.4 [19]
2,5-Dichloropyridine	-1166.200417	71.5 ± 2.2 [19]
2,6-Dichloropyridine	-1166.203496	71.4 ± 2.2 [19]
3,5-Dichloropyridine	-1166.197590	82.2 ± 2.1 [19]
Pyrimidine	-263.915002	195.7 ± 1.4 [17]
2-Chloropyrimidine	-723.078844	155.1 ± 1.6 [19]
2,4-Dichloropyrimidine	-1182.243354	125.6 ± 2.2 [17]
4,6-Dichloropyrimidine	-1182.244877	126.0 ± 2.2 [17]
2,4,6-Trichloropyrimidine	-1641.407011	85.8 ± 1.3 [19]
2,4,5,6-Tetrachloropyrimidine	-2100.562885	86.7 ± 2.1 [19]
Benzoic acid	-420.210294	-294.0 ± 2.2 [17]
2-Chlorobenzoic acid	-879.365005	-304.2 ± 0.7 [20]
3-Chlorobenzoic acid	-879.373326	-321.8 ± 3.7 [20]
4-Chlorobenzoic acid	-879.373923	-324.8 ± 3.7 [20]
1-Naphthalenecarboxylic acid	-573.594610	-223.1 ± 1.0 [17]
2-Naphthalenecarboxylic acid	-573.599298	-232.5 ± 1.7 [17]
2-Pyridinecarboxylic acid	-436.250294	-243.0 ± 2.6 [21]
3-Pyridinecarboxylic acid	-436.247160	-232.6 ± 1.5 [17]
4-Pyridinecarboxylic acid	-436.246034	-234.8 ± 4.7 [21]
Fenclorim	-1412.917444	--
Clopyralid	-1354.567694	--

Table S7. Working reactions and computed enthalpies of reaction, $\Delta_r H^o_m$, and formation, $\Delta_f H^o_m$, of fenclorim in the gaseous phase, at T = 298.15 K.

Reaction		$\Delta_r H^o_m$ / $kJ \cdot mol^{-1}$	$\Delta_f H^o_m(g)$ / $kJ \cdot mol^{-1}$
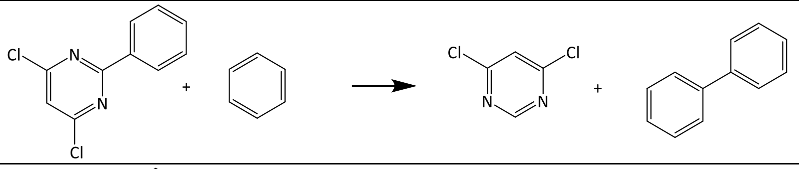	(1)	17.06<br>21.05 [a]	206.34<br>201.95 [a]
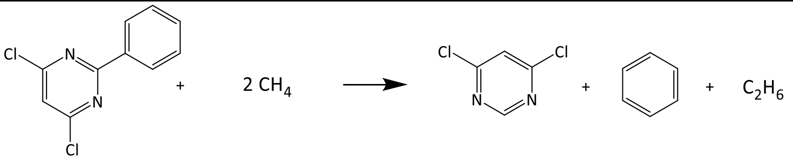	(2)	72.15<br>76.15 [a]	201.75<br>197.35 [a]

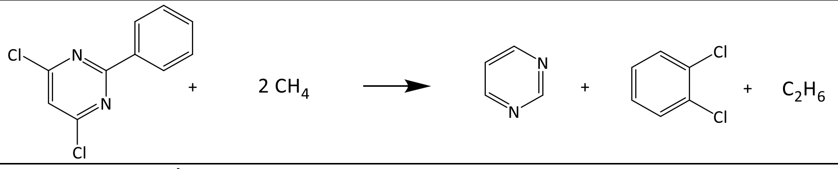	(3)	85.38<br>79.63 [b]<br>79.98 [c]	205.52<br>206.77 [b]<br>203.22 [c]
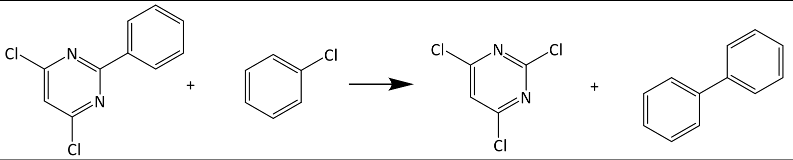	(4)	22.25	191.85
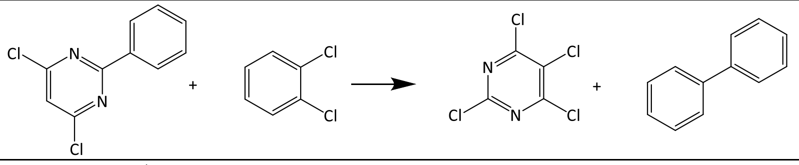	(5)	34.98<br>40.73 [b]<br>40.38 [c]	201.82<br>200.57 [b]<br>204.12 [c]
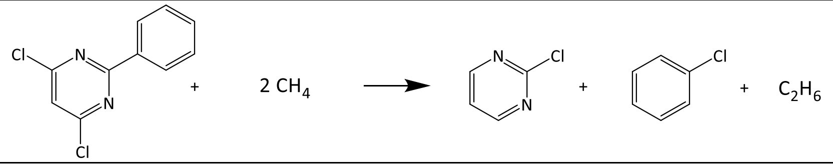	(6)	77.19	194.91
Mean value[d] / kJ.mol^{-1}			**201.35 ± 2.92**

[a]Reactions (1 and 2) using the isomer 2,4-dichloropyrimidine. [b]Reactions (3 and 5) using the isomer *m*-dichlorobenzene. [c]Reactions (3 and 5) using the isomer *p*-dichlorobenzene. [d]The uncertainty assigned correspond to the expanded uncertainty determined from the estimated standard deviation of the mean for the 12 reactions and the coverage factor $k = 2.20$ (0.95 level of confidence).

Table S8. Working reactions and computed enthalpies of reaction, $\Delta_r H_m^o$, and formation, $\Delta_f H_m^o$, of clopyralid in the gaseous phase, at $T = 298.15$ K.

**Reaction**		$\frac{\Delta_r H_m^o}{kJ \cdot mol^{-1}}$	$\frac{\Delta_f H_m^o(g)}{kJ \cdot mol^{-1}}$
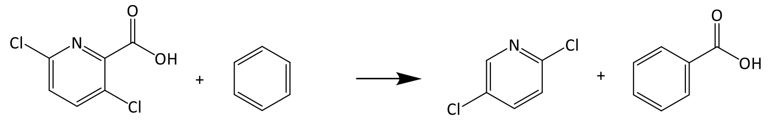	(1)	-20.62<br>-16.19 [a]<br>-28.70 [b]<br>-13.20 [c]	-284.78<br>-271.21 [a]<br>-276.80 [b]<br>-281.50 [c]
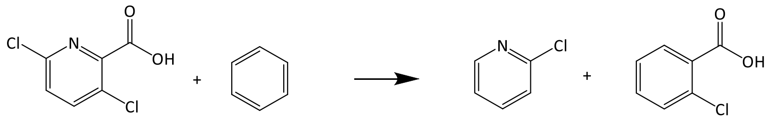	(2)	-3.26<br>-25.11 [d]<br>-26.68 [e]	-279.34<br>-275.09 [d]<br>-276.52 [e]
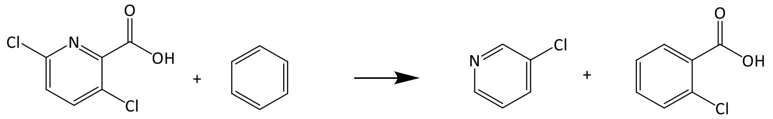	(3)	-4.86<br>-16.98 [d]<br>-18.55 [e]	-284.36<br>-280.12 [d]<br>-281.55 [e]
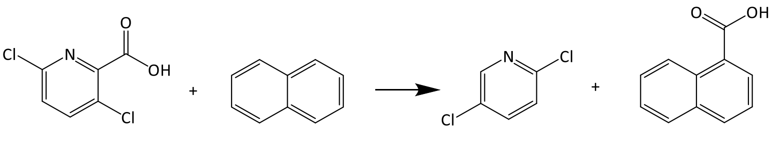	(4)	-3.46<br>-5.04 [a]<br>-17.54 [b]<br>-2.04 [c]	-292.44<br>-278.86 [a]<br>-284.46 [b]<br>-289.16 [c]
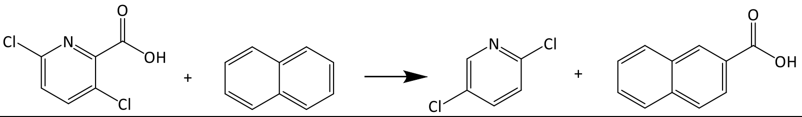	(5)	-21.77<br>-17.34 [a]<br>-29.85 [b]	-289.53<br>-275.96 [a]<br>-281.55 [b]

		-14.35 [c]	-286.25 [c]
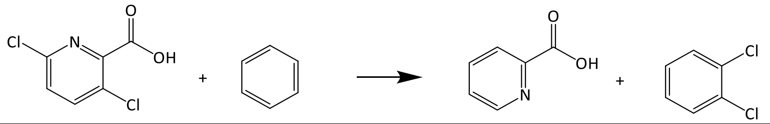	(6)	-19.52<br>-25.27 [f]<br>-24.92 [g]	-276.18<br>-274.93 [f]<br>-278.48 [g]
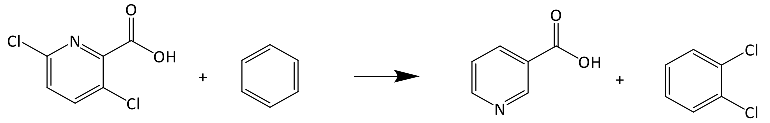	(7)	-11.29<br>-17.04 [f]<br>-16.69 [g]	-274.00<br>-272.76 [f]<br>-276.31 [g]
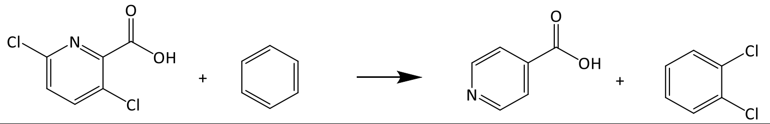	(8)	-8.34<br>-14.09 [f]<br>-13.73 [g]	-279.16<br>-277.91[f]<br>-281.47[g]
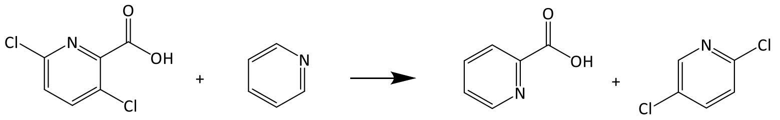	(9)	-25.17<br>-20.74 [a]<br>-33.25 [b]<br>-17.75 [c]	-286.73<br>-273.16 [a]<br>-278.75 [b]<br>-283.45 [c]
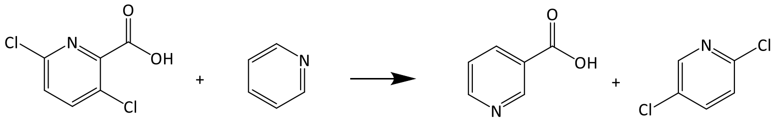	(10)	-16.94<br>-12.52 [a]<br>-25.02 [b]<br>-9.52 [c]	-284.56<br>-270.98 [a]<br>-276.58 [b]<br>-281.28 [c]
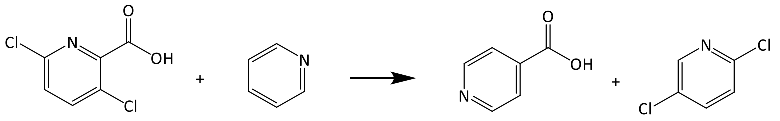	(11)	-13.98<br>-9.56 [a]<br>-22.07 [b]<br>-6.56 [c]	289.72<br>-276.14 [a]<br>-281.73 [a]<br>-286.44 [c]
Mean value[h] / kJ.mol^{-1}			**-280.26 ± 1.72**

[a]Reaction (1, 4, 5, 9, 10 or 11) using the isomer 2,3-dichloropyridine. [b]Reactions (1, 4, 5, 9, 10 or 11) using the isomer 2,6-dichloropyridine. [c]Reactions (1, 4, 5, 9, 10 or 11) using the isomer 3,5-dichloropyridine.[d]Reactions (2 or 3) using the isomer *m*-chlorobenzoic acid. [e]Reactions (2 or 3) using the isomer *p*-chlorobenzoic acid. [f]Reactions (6, 7 or 8) using the isomer *m*-chlorobenzene. [g]Reactions (6, 7 or 8) using the isomer *p*-chlorobenzene. [h]The uncertainty assigned correspond to the expanded uncertainty determined from the estimated standard deviation of the mean for the 39 reactions and the coverage factor $k = 2.02$ (0.95 level of confidence).

References

1. Kwon, E.; Kim, J.; Kang, G.; Kim, T.H. Crystal structure of fenclorim. *Acta Cryst*. **2015**. *E71*, o714.

2. Hamazaki, H.; Hosomi, H.; Takeda, S.; Kataoka, H.; Ohba, S. 2-Pyridinecarboxylic Acid. *Acta Cryst.* **1998**, *C54*, IUC9800049.

3. https://www.lookchem.com/Fenclorim/

4. https://www.chemsrc.com/en/cas/3740-92-9_670309.html

5. https://www.agropages.com/agrodata/Detail-602.htm

6. https://hybris-static-assets-production.s3-eu-west-1.amazonaws.com/sys-master/pdfs/hd0/ha3/9684274741278/EN_ST-WB-MSDS-2213897-1-1-1.PDF

7. Yaws, C.L. Thermophysical Properties of Chemicals and Hydrocarbons. Elsevier **2014**.

8. https://www.matrixscientific.com/043477.html

9. https://www.chemicalbook.com/ChemicalProductProperty_EN_CB6187251.htm

10. https://www.agropages.com/agrodata/Detail-602.htm

11. https://pubchem.ncbi.nlm.nih.gov/compound/77338

12. http://www.chemspider.com/Chemical-Structure.69755.html

13. http://sitem.herts.ac.uk/aeru/ppdb/en/Reports/296.htm

14. https://pubchem.ncbi.nlm.nih.gov/compound/Clopyralid

15. http://sitem.herts.ac.uk/aeru/ppdb/en/Reports/169.htm

16. http://www.molmall.net/product/100090

17. Pedley, J.B. Thermochemical Data and Structures of Organic Compounds Thermodynamics, Research Center, College Station, Texas**, 1994**.

18. Roux, M.V.; Temprado, M.; Chickos, J.S.; Nagano. Y.Critically evaluated thermochemical properties of polycyclic aromatic hydrocarbons, *J. Phys. Chem. Ref. Data* **2008**, *37*, 1855-1996.

19. Ribeiro da Silva, MA.V.; Amaral, L.M.P.F.; Gomes J.R.B. *J. Phys. Chem. B* **2007**, *111*, 792-799.

20. Sabbah, R.; Rojas Aguilar, A., Etude thermodynamique des trois isomeres de l'acide chlorobenzoique. Partie II, *Can. J. Chem.*, **1995**, *73*, 1538-1545.

21. Ribeiro da Silva, M.D.M.C.; Matos, M.A.R.; Vaz, M.C.; Santos, L.M.N.B.F.; Pilcher, G.; Acree Jr., W.E.; Powell, J.R. Enthalpies of combustion of the pyridine N-oxide derivatives: 4-methyl-, 3-cyano-, 4-cyano-, 3-hydroxy-, 2-carboxy-, 4-carboxy-, and 3-methyl-4-nitro, and of the pyridine derivatives: 2-carboxy-, and 4-carboxy-. The dissociation enthalpies of the N-O bonds. *J. Chem. Thermodyn.* **1998**, *30*, 869–878.
